# Socioecological drivers of injuries and aggression in female and male rhesus macaques (*Macaca mulatta*)

**DOI:** 10.1007/s00265-025-03587-3

**Published:** 2025-03-28

**Authors:** Melissa A. Pavez-Fox, Erin R. Siracusa, Samuel Ellis, Clare M. Kimock, Nahiri Rivera-Barreto, Josue E. Negron-Del Valle, Daniel Phillips, Angelina Ruiz-Lambides, Noah Snyder-Mackler, James P. Higham, Delphine De Moor, Lauren J. N. Brent

**Affiliations:** 1 Centre for Research in Animal Behaviour, University of Exeter, Exeter, EX4 4QG UK; 2 Department of Psychology and Neuroscience, University of St Andrews, St Andrews, KY16 9JP UK; 3 Department of Anthropology, New York University, New York, NY 10003 USA; 4 Department of Psychology, Nottingham Trent University, Nottingham, NG1 4FQ UK; 5 Caribbean Primate Research Center, University of Puerto Rico, San Juan, Puerto Rico 00936-5067 USA; 6 Center for Evolution and Medicine, Arizona State University, Temple, AZ 85281 USA; 7 School of Life Sciences, Arizona State University, Temple, AZ 85281 USA; 8 School for Human Evolution and Social Change, Arizona State University, Temple, AZ 85281 USA

**Keywords:** Social organization, Competition, Aggression, Injuries, Rhesus macaques

## Abstract

**Abstract:**

Competition over access to resources, such as food and mates, is one of the major costs associated with group living. Two socioecological factors believed to drive the intensity of competition are group size and sex ratio. However, empirical evidence linking these factors to physical aggression and injuries is scarce. Here, we leveraged 10 years of data from free-ranging female and male rhesus macaques to test whether group size and adult sex ratio predicted the risk of inter and intrasexual aggression, as well as injury risk. We found evidence for an optimal group size at which the risk of intragroup aggression was minimized for both sexes. Despite male-male aggression being lowest in mid-sized groups, males in smaller groups experienced higher injury risk, suggesting within-group aggression might not be the main cause of male injury. Additionally, we found that sex ratio influenced aggression, but not injury risk. Specifically, female aggression toward other females was heightened during the birth season when groups had fewer available males, suggesting either female competition for male friends or exacerbated female-female competition due to the energetic costs of lactation. Male aggression towards females was higher in female-biased groups during the birth season and in male-biased groups during the mating season, which could reflect male competition with females over feeding opportunities and male coercion of females, respectively. Together, these findings provide insights into fitness costs (i.e., injury risk) of inter and intrasexual competition in primates in relation to key aspects of social organization.

**Significance statement:**

While theory suggests that group size and sex ratio influence competition, studies linking these factors to aggression and injury rates are limited. Using long-term data on demography, aggression, and injury from a group-living primate, we show that both males and females experience aggression less often at intermediate group sizes. However, males in smaller groups faced higher injury risks. Although sex ratio did not predict injury risk, it did influence intra- and intersexual aggression, with patterns varying by reproductive season. Overall, our findings provide insights into how competition shapes intra and intersexual dynamics in relation to aspects of social organization.

**Supplementary Information:**

The online version contains supplementary material available at 10.1007/s00265-025-03587-3.

## Introduction

Competition over access to resources is an important selective pressure for the evolution of group living. By forming groups, animals can gain advantages such as higher success at locating food, more and easily accessible mating opportunities, decreased predation risk and cooperative defense of resources (van Schaik and van Hooff 1983; Jarvis et al. 1998; Ratcliffe and ter Hofstede 2005; Silk 2007). However, life in groups can also be associated with major costs for individuals as a result of competition with conspecifics where food and mates are the key limiting resources (van Schaik and van Hooff 1983; Terborgh and Janson 1986; Janson and Goldsmith 1995). Intense competition in the form of physical aggression can substantially impact an individual’s health by increasing their risk of injury (Vogel et al. 2007; Feder et al. 2019). Injuries may indirectly impact reproductive success as animals may need to divert energetic resources to healing (Archie et al. 2014), and can directly impact survival in the case of fatal aggression (Chilvers et al. 2005; Pavez-Fox et al. 2022). Given the fitness costs of injuries, animals are expected to refrain from engaging in physical aggression unless necessary when resources are limiting or very valuable (Hammerstein 1981). Two socioecological factors have been hypothesized to drive the intensity of competition: group size and the operational sex ratio (Kvarnemo and Ahnesjo 1996; Chapman and Chapman 2000).

Group size can determine the intensity of competition within and between groups over food and territories. On the one hand, group size is often limited by food availability, as a larger group depletes resources faster (Chapman and Chapman 2000). For example, in macaques (*Macaques* spp*.*) (Balasubramaniam et al. 2014; Heesen et al. 2014), red colobus (*Procolobus badius*) (Gillespie and Chapman 2001), olive baboons (*Papio anubis*) (Patterson et al. 2021), mountain gorillas (*Gorilla beringei beringei*) (Seiler and Robbins 2020), tammar wallabies (*Macropus eugenii*) (Blumstein et al. 1999) and guanacos (*Lama guanicoe*) (Marino 2010)), animals in larger groups experienced more intense within-group competition for food compared to those in smaller groups. On the other hand, when feeding areas can be monopolized and are extensive enough to sustain entire groups, larger groups have a numerical advantage over smaller groups, which can be beneficial for the collective defense of the territories holding such resources (Cheney and Seyfarth 1987; McComb et al. 1994). For instance, larger groups of several nonhuman primates (Balasubramaniam et al. 2014; Majolo et al. 2020), banded mongooses (*Mungos mungo*) (Thompson et al. 2017) and meerkats (*Suricata suricatta*) (Dyble et al. 2019) are more likely to win between-group encounters versus smaller groups. As a result, the interplay between the costs of increased within-group competition in larger groups and the benefits that entails for between-group competition, have led to the suggestion of optimal group sizes at intermediate number of individuals (Pride 2005; Markham et al. 2015) in which the benefits gained from holding territories might compensate for the costs of feeding competition within the group.

In addition to food, group-living animals also compete for access to mates. Mating competition has been suggested to be driven primarily by the relative availability of sexually active males and females in a group (the operational sex ratio). When the operational sex ratio is skewed, theory predicts there will be higher competition among the more abundant sex over access to the less abundant sex (Emlen and Oring 1977; Clutton-Brock and Parker 1992; Kvarnemo and Ahnesjo 1996). For instance, in gorillas (*Gorilla* spp*.*), female-female competition for males in the form of aggression was higher in groups with a female-biased sex ratio (Smit and Robbins 2024). Similarly, in vervet monkeys (*Chlorocebus pygerythrus*), male-male fights were more frequent in groups with male-skewed operational sex ratios (Hemelrijk et al. 2020). However, when the operational sex ratio is too skewed and the costs associated with aggression are too high, a reduction in intrasexual competition might be favored leading to the emergence of alternative strategies including indirect mating competition without physical aggression (e.g*.,* sperm competition, endurance, copulatory plugs, courtship displays) and/or inter-sexual aggression (i.e*.,* coercion) (Rankin et al. 2011; Weir et al. 2011; Higham and Maestripieri 2014). For example, a female-biased sex ratio predicted a higher frequency of display of female courtship behavior in reindeer (*Rangifer tarandus*) (Driscoll et al. 2022), while a male-biased sex ratio was associated with more frequent male–female attacks in lizards (*Lacerta vivipara*) (Le Galliard et al. 2005). As a consequence, the operational sex ratio might not only determine costs derived from intrasexual mating competition but also from inter-sexual aggression.

Although drivers of competition in group-living animals have been well established theoretically (Trivers 1972; van Schaik and van Hooff 1983), there is still scarce empirical evidence on how these factors affect physical aggression and injuries. Quantifying the consequences of contest competition has proven difficult in most wild systems where observed injuries or body damage can be caused by predators and may not be the direct result of competition with conspecifics. Furthermore, given the differences in life history between the sexes, the costs and drivers of competition are often studied separately for males and females, although there is growing evidence that competition can also result in sexual conflict (Baniel et al. 2017; Davidian et al. 2022; Smit et al. 2022).

Here, we tested for sex-specific effects of group size and adult sex ratio, a proxy of operational sex ratio, on the occurrence of contact aggression and injuries in free-ranging female and male rhesus macaques living in Cayo Santiago, Puerto Rico. Rhesus macaques live in multi-female multi-male societies where females are philopatric and males disperse at sexual maturation (Bernstein 2005). Females form strict despotic dominance hierarchies where rank is maternally inherited (Chikazawa et al. 1979). Males, instead, acquire rank via a queuing system where group tenure determines their social status (Manson 1995; Kimock et al. 2022). Rhesus macaques have a polygynandrous mating system with high synchrony in the fertile phases of the females, reducing the monopolization potential for males (Dubuc et al. 2011). As a consequence, male rhesus often rely more on indirect forms of competition, such as sperm competition, endurance rivalry, sneaky copulations and female coercion (Manson 1994; Higham et al. 2011; Higham and Maestripieri 2014), rather than direct male-male conflict (Kimock et al. 2022). There are no predators on the island, allowing us to isolate the role of competition in shaping aspects of rhesus macaque social organization. Social groups are naturally formed and can vary in size from 26 to nearly 300 adults. Long-term behavioral observations have been collected in several of these groups allowing us to pair injury data with the occurrence of aggressive events to explore sex differences in the identity of the victims and aggressors. Injuries are known to be primarily caused by conspecifics and have severe consequences for male and female survival (Pavez-Fox et al. 2022), which provides the opportunity to test the fitness-related costs of competition.

**Fig. 1:**
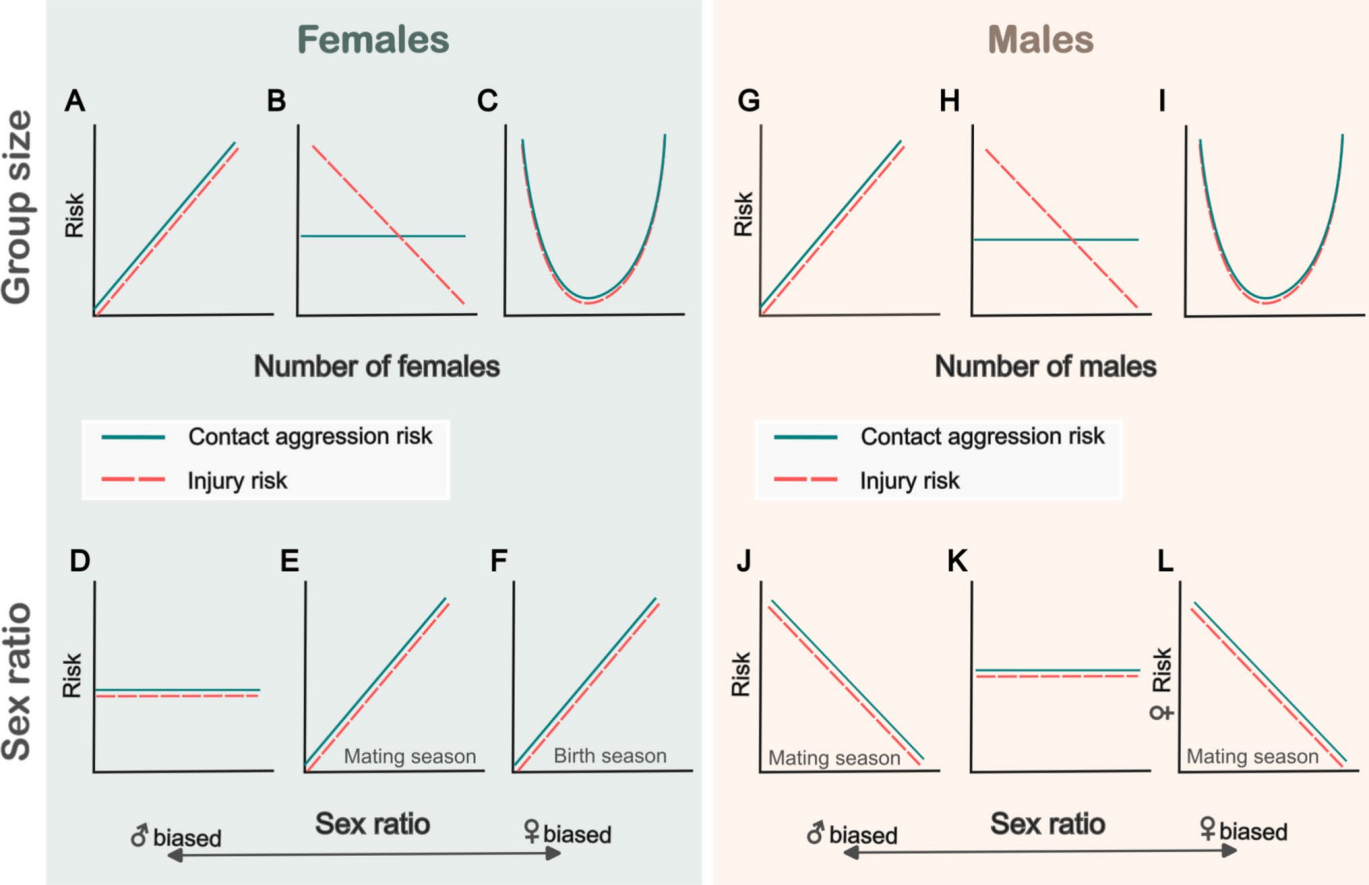
Potential primary drivers of contact aggression and injury risk in female and male rhesus macaques. Predictions for the following hypothesized drivers: (**A**), (**G**) “within-group competition” hypothesis; (**B**), (**H**) “between-group competition” hypothesis; (**C**), (**I**) “optimal group size” hypothesis; (**D**) “no competition for males” hypothesis; (**E**) “competition for males” hypothesis; (**F**) “competition for male friends” hypothesis; (**J**) “direct competition for females” hypothesis; (**K**) “indirect competition” hypothesis and (**L**) “female coercion” hypothesis. Patterns for injuries are depicted in red dashed lines and contact aggression is in solid blue lines. The y-axis in all the cases represents the risk of injuries and contact aggression for females (left square) and for males (right square) with the exception of Fig. 1L where male coercion is expected to influence injury and contact aggression risk for females

Socioecology is an area of active research where there is not necessarily a clear consensus on how group size and sex ratio should influence costs of competition. In this manuscript, we aim to test several competing hypotheses to better understand the role these socioecological factors may play in aggression and injury risk. We begin by examining the socioecological predictors of injuries and contact aggression in females. Given the high energetic costs of gestation and lactation, the fitness of females is believed to be determined mostly by access to food (Trivers 1972). Because food constitutes a defendable and limiting high-quality resource, contest competition among females can be expected both within and between groups (Wrangham 1980; van Schaik 1989; Koenig et al. 2013). Although our study population is food provisioned, females have been shown to compete over feeding and drinking stations (Balasubramaniam et al. 2014). Access to feeders can be monopolized by a subset of individuals, thus we can expect higher within-group competition among females living in larger groups, which would translate into higher injury and contact aggression risk in larger groups (“within-group competition” hypothesis, prediction Fig. 1A). Alternatively, it has also been suggested that cooperation among kin might be an evolutionary driver for females to live together (Wrangham et al. 1993). Female rhesus macaques are philopatric and groups are composed of a mixture of unrelated and related females, the latter of which promotes the formation of social bonds (Widdig et al. 2006) and could favor the cooperative defense of resources (Wrangham 1980; Sterck et al. 1997; Young and Bennett 2013). If this is the case, we expect that group size would not predict female-female contact aggression risk within groups but as group size increases, females would experience lower injury risk due to advantages in between-group competition. Although we do not have behavioral data from between-group encounters, we expect that injury records, which were recorded ad libitum, can provide an approximation of costs derived from both within and between-group competition (“between-group competition” hypothesis, prediction Fig. 1B). If the hypothesized trade-off among within and between group competition (Pride 2005; Markham et al. 2015) applies to female rhesus macaques, we expect a U-shape relationship between group size and female injury and, between group size and female-female contact aggression risk where risks are the lowest at intermediate group sizes (“optimal group size” hypothesis, prediction Fig. 1C).

When it comes to mating competition, female mammals are not commonly believed to compete aggressively over males. This is because females can share male paternity and the costs of engaging in physical aggression might be too high to sustain, given the energetic costs of reproduction (Davidian et al. 2022). Further, competition against kin in female-philopatric societies like rhesus macaques might further reduce the incentive of using physical aggression (Young and Bennett 2013). If this is the case for female rhesus, the sex ratio of a group should not predict competition among females. That is, we expect no relationship between sex ratio and female injury and contact aggression risk (“No competition for males” hypothesis, prediction Fig. 1D). However, growing evidence suggests that female mammals can also engage in competition over mates, possibly driven by desired male attributes such as resource holding potential (Stockley and Bro-Jørgensen 2011) or genetic quality (Rosvall 2011), as well as a means to suppress reproduction of other females (Young et al. 2006) or to ensure male protection for their offspring (Clutton-Brock and McAuliffe 2009; Baniel et al. 2018a). If female rhesus macaques compete for mating partners or attempt to suppress conception in other females, we expect that as the sex ratio becomes more female biased, female injury and contact aggression risk would increase. Further, we expect to observe this pattern only during the mating season which is when copulations and conceptions occur (“competition for males” hypothesis, prediction Fig. 1E). In contrast, if females try to gain male protection for their offspring, one strategy is to form male–female long-term affiliative relationships (‘friendships’) which often precede the mating period (Baniel et al. 2016; Stadele et al. 2021). If females compete for male friends, we expect to see the same pattern of female injuries and contact aggression as predicted for the mating season, but during the birth season (“competition for male friends” hypothesis, prediction Fig. 1F).

In males, the drivers of aggression and injuries are believed to be mostly related to the access and monopolization of females (Trivers 1972). In this regard, living in larger groups might provide males with a numerical advantage to defend females against rival males from other groups (Cowlishaw 1995; Scarry 2013; Majolo et al. 2020; Smith et al. 2022). If males engage in contact aggression predominantly in the context of between-group conflicts, we expect that group size would not predict male-male contact aggression risk within the group but as the group becomes larger, males would have lower injury risk due to advantages in between-group competition (“between-group competition” hypothesis, prediction Fig. 1H). However, when male competitive outcome is based on their body condition, as is the case for rhesus macaques (i.e*.,* endurance rivalry) (Higham and Maestripieri 2014), food can also become a limiting factor. If male rhesus macaques engage in direct contests over food, we expect that male injury and contact aggression risk would increase with group size as a result of enhanced within-group competition (“within-group competition” hypothesis, prediction Fig. 1G). Given these two scenarios, it might be possible that there is an optimal group size for males in which the costs of within-group competition are minimized, while the benefits for between-group competition are maintained. If this is the case, we expect male injury risk and aggression rates to be lowest at an intermediate group size (“optimal group size” hypothesis, prediction Fig. 1I).

Finally, the operational sex ratio theory predicts that in groups with skewed sex ratios, individuals of the more abundant sex face higher costs of competition over mates (Kvarnemo and Ahnesjo 1996). This might be particularly true for male mammals in uniparous species, like rhesus macaques, where paternity cannot be shared. As a result, we would expect that in more male-biased groups, the intensity of male direct competition increases. That is, males living in groups with male-biased sex ratios are expected to have higher injury and contact aggression risk than males living in groups with female-biased sex ratios, particularly during the mating season (“direct competition for females” hypothesis, prediction Fig. 1J). Alternatively, males may also compete indirectly via endurance, sperm competition and sneaky copulations. Indeed, these strategies have been observed in male rhesus macaques (Higham and Maestripieri 2014; Kimock et al. 2022), suggesting that we could also expect a null relationship between sex ratio and male injury or contact aggression risk (“indirect competition” hypothesis, prediction Fig. 1K). Lastly, one strategy often adopted by males to reduce costs of retaliation when competing against other males is to redirect aggression towards females (Rankin et al. 2011; Weir et al. 2011; Davidian et al. 2022). Given that rhesus macaques have a moderate degree of sexual size dimorphism, males have the physical advantage to force females into copulation or to stop copulations with other males (i.e*.,* mate guarding) (Manson 1994). If this strategy is common, we would expect that in groups with male-biased sex ratios male–female contact aggression rates would be higher, which might also be reflected in higher injury risk for females in those groups during the mating season (“female coercion” hypothesis, prediction Fig. 1L).

## Methods

### Study subjects

Study subjects were free-ranging male and female rhesus macaques living on Cayo Santiago island, Puerto Rico. The island is home to a population of ∼ 1800 individuals living in 6 to 12 multi-male multi-female naturally formed social groups. The Cayo Santiago field station is managed by the Caribbean Primate Research Center (CPRC), which monitors the population daily and maintains the long-term (> 75 years) demographic database including data on births, deaths and social group membership for all animals (Kessler and Rawlins 2016). Macaques are individually identified based on tattoos located on their chest and legs. Animals have ad libitum access to food and water, the island is free of predators and there is no regular medical intervention for sick or wounded individuals. Here we included data on male and female macaques that were alive between 2010 and 2020. We focused on individuals aged 4 years and above (age range: 4—28 years), as animals of both sexes have typically reached sexual maturity at that age (Bercovitch and Berard 1993; Zehr et al. 2005). We restricted our sample to animals belonging to social groups for which we had data on injury occurrence and agonistic behavioral observations (*n* = 6 social groups). Note that it was not possible to record data blind because our study involved focal animals in the field*.*

### Observation of injuries

Since 2010, the CPRC staff have been collecting opportunistic observations on the incidence and recovery from injuries during the daily monitoring of social groups for demographic purposes. Data collection was mainly carried out by the veterinary technician from Monday to Friday between 7:30 and 14:00 and was complemented by information from other experienced staff. If an individual was observed to be wounded or displaying signs of injury (e.g*.,* limping) the staff member recorded the individual ID and if the injury was visible, the type of injury (e.g*.,* puncture, scratch), the area of the body affected, whether the injury was recent or old based on the presence of scars, and if possible, an estimate of the wound size. Records for each individual were updated every time the observers encountered the wounded individuals during the daily census. Here we included all records for visible injuries including bites, scratches, abrasions and cuts along with other more severe injuries such as exposed organs and fractures. We decided to exclude cases where injuries could be inferred but were not observed, such as limping or abscesses, as these could also be caused by infection unrelated to injury. We excluded injury records from two full years (2015 and 2016), a period for which the veterinary technician was not regularly at the field site, which may have led to biases in the few groups sampled during those years. Our sample consisted of 908 injuries collected from September 2010 to April 2020 on 521 unique individuals (*n* females = 267, *n* males = 254).

### Collection of aggression data

Behavioral data were collected by two experienced research assistants using focal samples based on a previously established ethogram (Brent et al. 2014). Inter-observer reliability was assessed using the Noldus Observer software; an agreement above 85% was considered reliable. We collected data from twenty different group years (group F 2010–2017, group HH 2014 and 2016, group KK 2015 and 2017, group R 2015 and 2016, group S 2011 and 2019, group V 2015–2019). Across the 10 years of study, two external events in 2018 and 2020—Hurricane Maria and the COVID-19 pandemic, respectively—precluded the collection of focal data. These years were excluded from the aggression analyses. In total, this resulted in data for 748 adult individuals (422 females and 326 males) whose ages ranged between 4–28 years old (mean = 10.7). Behavioral data were collected using 10-min (17 group years) or 5-min (3 group years) focal animal samples between 07:30 and 14:00 from Monday to Friday. We stratified sampling to ensure balanced data collection on individuals throughout the day and over the year (focal follows per individual/year: median = 26, SD = 13, range = 0—63). During focal sampling, dyadic agonistic encounters that involved the focal animal were recorded, along with the identity of the aggressor and victim. If the focal animal was involved in coalitionary aggression, the interaction was recorded as multiple dyadic events. Given that the purpose of our study was to use the aggression data to contextualize the occurrence of injuries, we considered only data on contact aggression (e.g., bites, hits), which is more likely to lead to an injury. From January 2010 to October 2019, we recorded 522 physical aggression events.

### Quantifying injury and contact aggression risk

The injury dataset included the 521 animals that were recorded injured in addition to 1001 uninjured animals (*n* uninjured females = 525, *n* uninjured males = 476). Uninjured individuals consisted of all sexually mature individuals who were alive during the period of study, i.e., between 2010 and 2020 excluding 2015 and 2016 to match data on injured animals. As our predictions were sex-specific, we split the injury dataset by sex, resulting in a total of 792 females and 730 males. We formatted the datasets in a way that each row represented a two-month interval period (i.e., bimonthly interval). We based this decision on the fact that the average recovery time for an injury was 43 days and the average time elapsed between consecutive injury records was 42.17 days. By formatting the data this way, we could be confident that injury records occurring in different bimonthly intervals were more likely to be independent (for details see SI: Pavez-Fox et al. 2022). An individual’s injury status during each bimonthly interval was coded as a binary variable where 1 = injured and 0 = uninjured.

The aggression dataset included the 748 male and female macaques for which focal data were collected. We also split this dataset by the sex of the focal animal resulting in 438 contact aggression events in a total of 422 females and 84 contact aggression events in a total of 326 males. We focused specifically on contact aggression received by the focal animal and depending on the question, we either included aggressive events that were received from same sex or opposite sex individuals. Each row represented a bimonthly interval to match the format of the injury data (focal follows per individual/bimonth: median = 6.8, SD = 4.5, range = 0—30). Given that an individual rarely received contact aggression more than once in a given bimonthly interval (Fig. S2), we coded an individual’s aggression status as binary, where 1 = aggressed and 0 = not aggressed.

### Determining group size, sex ratio and the reproductive season

Using demographic records, we computed group size as the number of adults (4 years and above) that were alive in a subject’s group in a given bimonthly interval. We specifically determined a group’s size at the middle of the interval (end of the first month), thus if an individual reached 4 years of age or died during the second month, this was only reflected in the following bimonthly interval. Depending on the prediction being tested, we used the number of females or males rather than group size as our predictor of interest, as these metrics better reflect intrasexual competition and were strongly correlated with group size (Fig. S3; Number of females: Pearson’s *R* = 0.94, *p* < 0.01, Number of males: Pearson’s *R* = 0.97, *p* < 0.01).

We computed the sex ratio as the number of females (4 years and above) per male in the subject’s group in a given bimonthly interval. Therefore, smaller numbers would indicate a male-biased sex ratio while larger numbers would indicate a female-biased sex ratio. As with group size, we determined a group’s sex ratio at the middle of the bimonthly interval. We determined the reproductive season following Hoffman et al. (2008). Briefly, we first computed the mean birth date ± 2 SD for each year. The start of the birth season was defined as the first birth date and the end, as the last birth date. The beginning of the mating season was determined by subtracting the gestation period of rhesus macaques (165 days; Silk et al. 1993) from the start of the birth season, and the end of the mating season was determined by subtracting the gestation period from the end of the birth season. If the middle of the bimonthly interval fell outside the mating season it was considered part of the birth season. The groups analyzed varied in size from 26 to 288 animals and sex ratios (*n* females/ *n* males) ranged from 0.5 to 4.5 (Fig. S1).

### Statistical analyses

We ran all the models in a Bayesian framework using the brms R Package (Burkner 2021). Therefore, evidence of an effect was determined based on the degree of overlap between the credible interval (CI) and zero (i.e., 89% non-overlapping reflecting evidence of an effect, Kruschke 2015). Given that all the dependent variables were coded as binary, models were fit using a Bernoulli distribution. All continuous predictors were z-scored. In all the models we included random intercepts for individual ID and for the specific bimonthly interval within the study period to account for repeated measures and population level effects through time, respectively. We assumed normal distributions for priors (mean = 0, SD = 1) and ran 10000 iterations in all the models. Model assumptions and posterior predictive checks were done using the’ppcheck ‘ built-in function from the brms package. Marginal effects were calculated using the emmeans R package (Lenth et al. 2018). We reported means as point estimates, standard error (SE) and 89% credible intervals of the posterior distribution. For marginal effects, we reported the median and the 89% highest posterior density interval (HPD).

### Effect of group size and sex ratio on female injury risk

To test predictions about female injury risk, we ran a single model where the dependent variable was a female’s injury status (0/1). To evaluate the “within-group” and “between-group” competition hypotheses we included a linear term for the number of females in the group (Fig. 1 predictions A and B, respectively). To examine the “optimal group size” hypothesis we additionally included a quadratic term for the number of females in the group (Fig. 1 prediction C). To assess the “no competition for males”, “competition for males” and “competition over male friends” hypotheses we also included an interaction between sex ratio and the reproductive season (Fig. 1 predictions D, E and F, respectively). Note that this interaction also addresses the “female coercion” hypothesis (Fig. 1 prediction L). If we did not find evidence for an effect of the quadratic term or the interaction term, we ran simplified models without those terms to determine the linear effects of our predictors of interest.

### Effect of group size and sex ratio on female-female contact aggression risk

As with the injury data, we tested all predictions for female aggression risk running a single model. The dependent variable was whether or not a female was aggressed by another female in a given bimonth (0/1). We included the same independent variables as the model for female injury risk with the addition of an offset term for sampling effort (i.e., number of hours that the female was focaled in that bimonthly interval). As above, we ran simplified models if there was no (or weak) evidence for quadratic or interactive effects.

### Effect of group size and sex ratio on male injury risk

To test predictions about male injury risk we ran a single model where the dependent variable was a male’s injury status (0/1). To evaluate the “within-group” and “between-group” competition hypotheses we included a linear term for the number of males in the group (Fig. 1 predictions G and H, respectively). To examine the “optimal group size” hypothesis we additionally included a quadratic term for the number of males in the group (Fig. 1 prediction I). To assess the “competition for females” and the”indirect competition” hypotheses, we included an interaction between sex ratio and the reproductive season (Fig. 1 predictions J and K, respectively). As above, we ran simplified models if there was no (or weak) evidence for quadratic or interactive effects.

### Effect of group size and sex ratio on male-male and male–female contact aggression risk

We tested all the predictions for male-male contact aggression risk by running a single model where the dependent variable was whether or not a male was aggressed by another male in a given bimonth 0/1). We included the same independent variables as the model for male injury risk.

To test the “female coercion” hypothesis (Fig. 1 prediction L), we used aggression data where the victims were females and the aggressors were males, such that the dependent variable reflected if a female had received aggression (0/1) from a male in a given bimonth. We ran a single model with the same structure as the models above (even though we did not have predictions for an effect of group size on male–female aggression) to get estimates for the effect of sex ratio on aggression risk independent of group size. That is, we included as independent variables a linear and a quadratic term for group size and an interaction between sex ratio and the reproductive season. Note that in this case group size includes both males and females, as the dependent variable relates to intersexual aggression.

## Results

The number of injuries in a group per bimonthly interval ranged from 0 to 12 in females (mean = 1.67, SD = 2) and from 0 to 13 in males (1.87 ± 2.37). Contact aggression rates per group and bimonth ranged from 0 to 0.18 aggressive events per hour for female-female aggression (0.04 ± 0.035), 0 to 0.14 per hour for male-male aggression (0.021 ± 0.03) and 0 to 0.31 per hour for male–female aggression (0.037 ± 0.045).

### Effect of group size and sex ratio on female injury risk

**Fig. 2:**
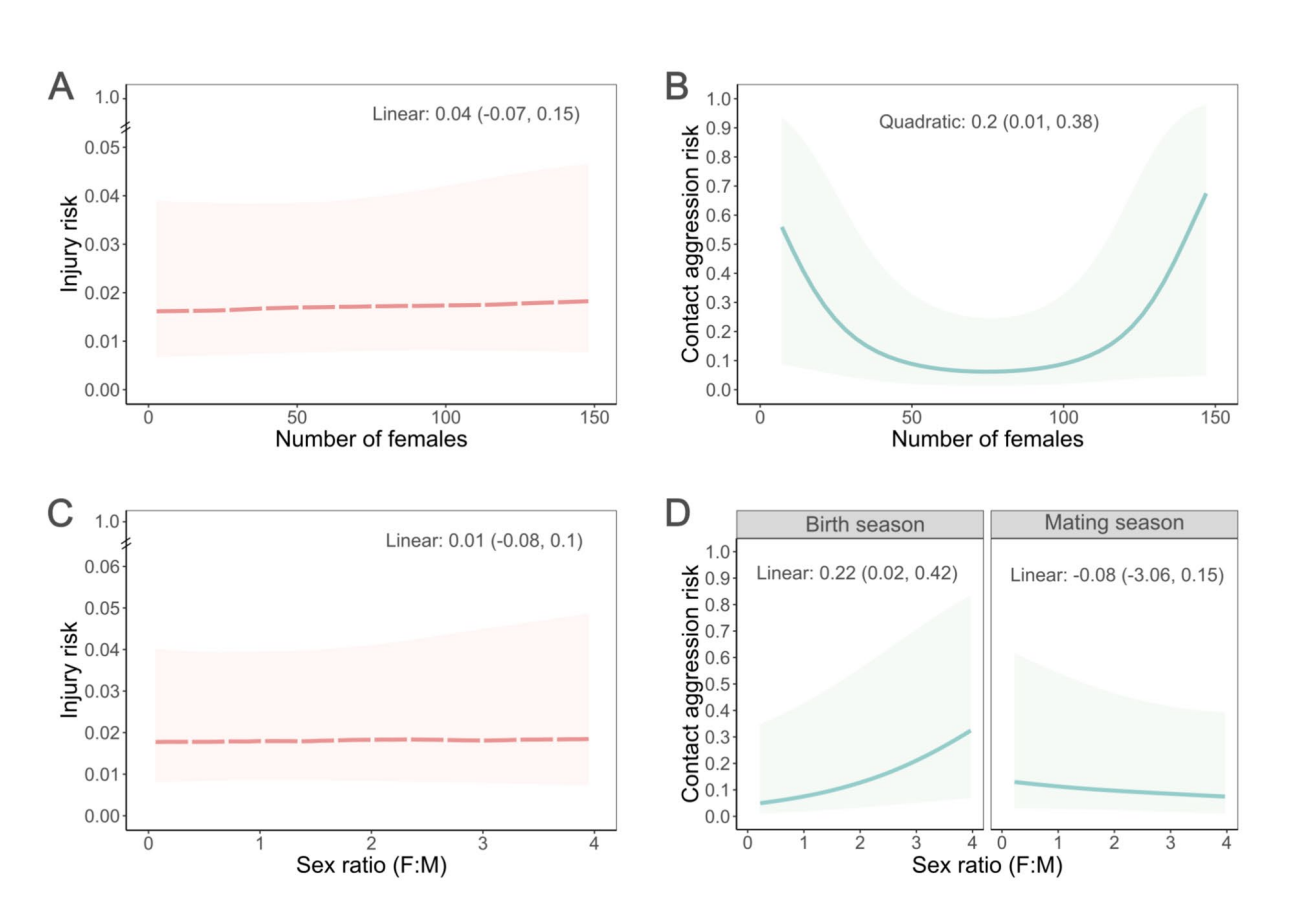
Effects of group size and sex ratio on female injury and contact aggression risk. Estimates for **(A)** injury risk and **(B)** female-female contact aggression risk as a function of group size (number of females in the group). **C)** Estimates of female injury risk as a function of sex ratio. **D)** Estimates of female-female contact aggression risk as a function of the reproductive season and sex ratio (proportion of females to males in the group). Lines represent the median and the shaded area the 89% CI from 100 draws of the posterior distribution (red and dashed line for injury risk, blue and solid line for aggression risk). Values along the top of the plots are the mean and the 89% CI (Figures **A**-**C**) or the median and 89% HPDI (Figure **D**) from the posterior distribution for the variable of interest

The number of females in a group did not predict a female’s probability of being injured (Fig. 2A). We did not find support for a linear (Log-Odds num fem = 0.04, SE = 0.07, 89% CI = −0.07, 0.15, Fig. 1 predictions A and B, Table S3) or quadratic (Log-Odds num fem2 = −0.07, SE = 0.06, 89% CI = −0.17, 0.03, Fig. 1 prediction C, Table S2) relationship between the number of females in a group and her risk of injury.

We did not find evidence for an effect of sex ratio on female’s injury risk, either as a main effect or in interaction with the reproductive season (Log-Odds sex ratio = 0.01, SE = 0.24, 89% CI = −0.08, 0.1, Fig. 1 prediction E, F and L, Table S3; Log-Odds sex ratio*season = 0.08, SE = 0.11, 89% CI = −0.1, 0.26, Fig. 1 prediction D, Table S1) (Fig. 2C).

### Effect of group size and sex ratio on female-female contact aggression

We found evidence for a positive quadratic relationship between the number of females in a group and the risk of female aggression (Log-Odds num fem2 = 0.2, SE = 0.11, 89% CI = 0.01, 0.38, Fig. 1. prediction C, Table S4). Females in smaller and larger groups had a higher risk of contact aggression than females in intermediate group sizes (Fig. 2B).

We found an effect of sex ratio on female-female contact aggression risk that was dependent on the reproductive season (Fig. 2D; Log-Odds sex ratio*season = −0.3, SE = 0.18, 89% CI = −0.59, −0.01; Table S4). During the birth season, females had higher risk of aggression when living in groups with a female-biased sex ratio compared to groups with a male-biased sex ratio (marginal effect: Log-Odds birth season = 0.22, 89% HPDI = 0.021, 0.43, Fig. 1 prediction F). Sex ratio did not predict female aggression risk during the mating season (marginal effect: Log-Odds mating season = −0.078, 89% HPDI = −3.06, 0.152, Fig. 1 prediction E).

### Effect of group size and sex ratio on male injury risk

**Fig. 3:**
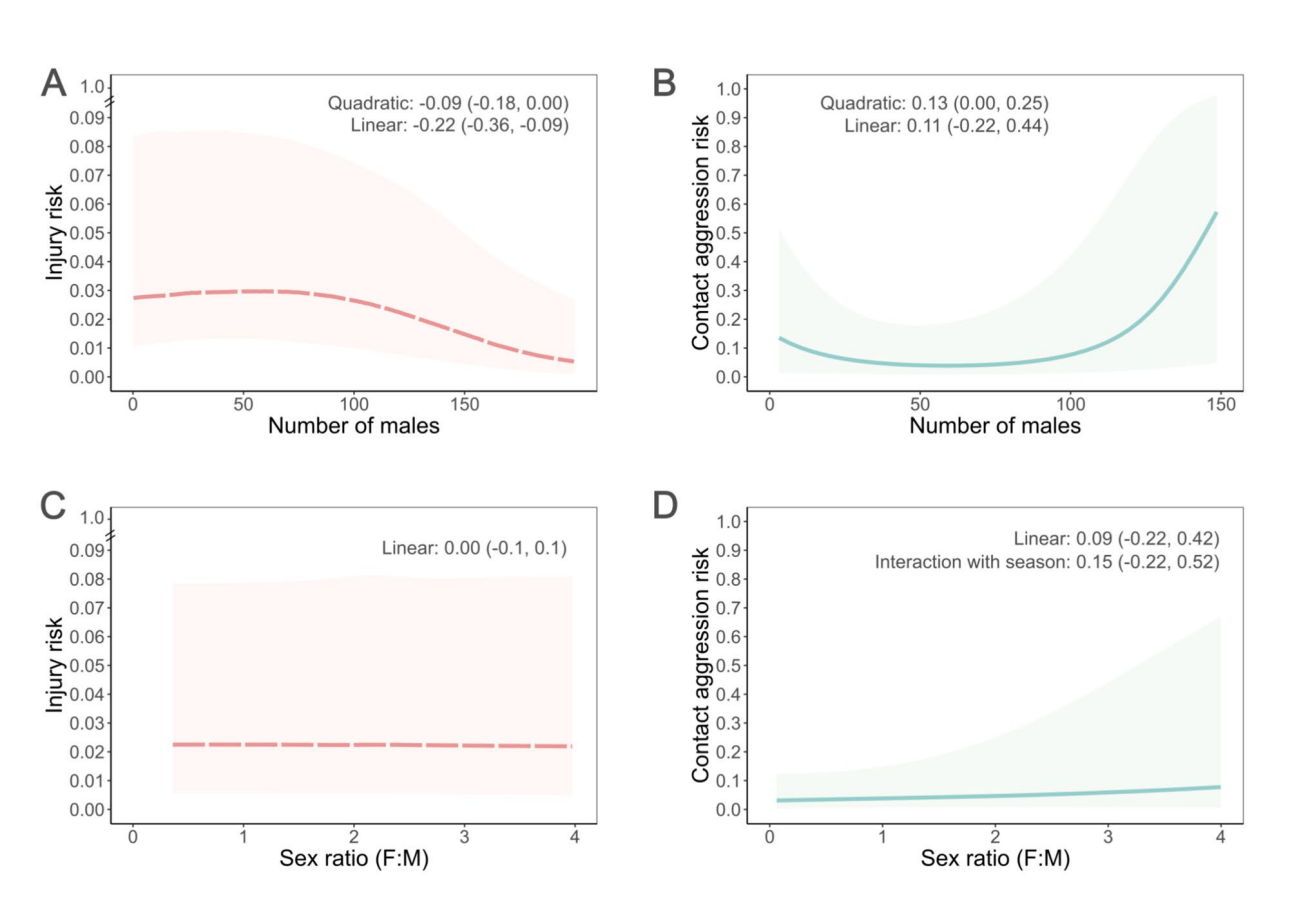
Effects of group size and sex ratio on male injury and contact aggression risk. Estimates for **(A)** injury risk and **(B)** male-male contact aggression risk as a function of group size (number of males in the group). Estimates for **(C)** injury risk **(D)** and male-male contact aggression risk as a function of sex ratio (proportion of females to males in the group). Lines represent the median and the shaded area the 89% CI from 100 draws of the posterior distribution (red and dashed line for injury risk, blue and solid line for aggression risk). Values along the top of the plots are the mean and the 89% CI from the posterior distribution for the variable of interest.

We found evidence for a negative linear relationship between the number of males in a group and male injury risk (Log-Odds num male = −0.22, SE = 0.08, 89% CI = −0.36, −0.09, Fig. 1 prediction H, Table S7) with a weak negative quadratic component (Log-Odds num male2 = −0.09, SE = 0.06, 89% CI = −0.18, 0.00; Table S6). Males living in larger groups were less likely to be injured than males living in smaller and intermediate size groups (Fig. 3A).

We did not find evidence that sex ratio predicted male injury risk either as a main effect (Log-Odds sex ratio = 0.03, SE = 0.07, 89% CI = −0.08, 0.14, Fig. 1 prediction K, Table S6) or in interaction with the reproductive season (Log-Odds sex ratio*season = −0.06, SE = 0.1, 89% CI = −0.23, 0.11, Fig. 1 prediction J, Table S5) (Fig. 3C).

### Effect of group size and sex ratio on male-male and male–female contact aggression

We found weak evidence for a positive quadratic relationship between group size and male-male contact aggression (Log-Odds num male2 = 0.13, SE = 0.08, 89% CI = 0.00, 0.23, Fig. 1 prediction I, Table S9). Males living in intermediate sized groups tended to have lower risk of contact aggression than males in smaller and larger groups, but the difference in effect seemed to be greater between intermediate and larger groups (Fig. 3B). We found no evidence for a linear effect of group size on male-male contact aggression risk (Log-Odds num male = 0.11, SE = 0.21, 89% CI = −0.22, 0.44, Fig. 1 prediction G and H, Table S10).

We did not find evidence for a relationship between sex ratio and male-male contact aggression risk, either as a main effect (Log-Odds sex ratio = 0.09, SE = 0.2, 89% CI = −0.22, 0.42, Fig. 1 prediction K, Table S10) or in interaction with the reproductive season (Log-Odds sex ratio*season = 0.15, SE = 0.23, 89% CI = −0.22, 0.52, Fig. 1 prediction J, Table S8) (Fig. 3D).

**Fig. 4:**
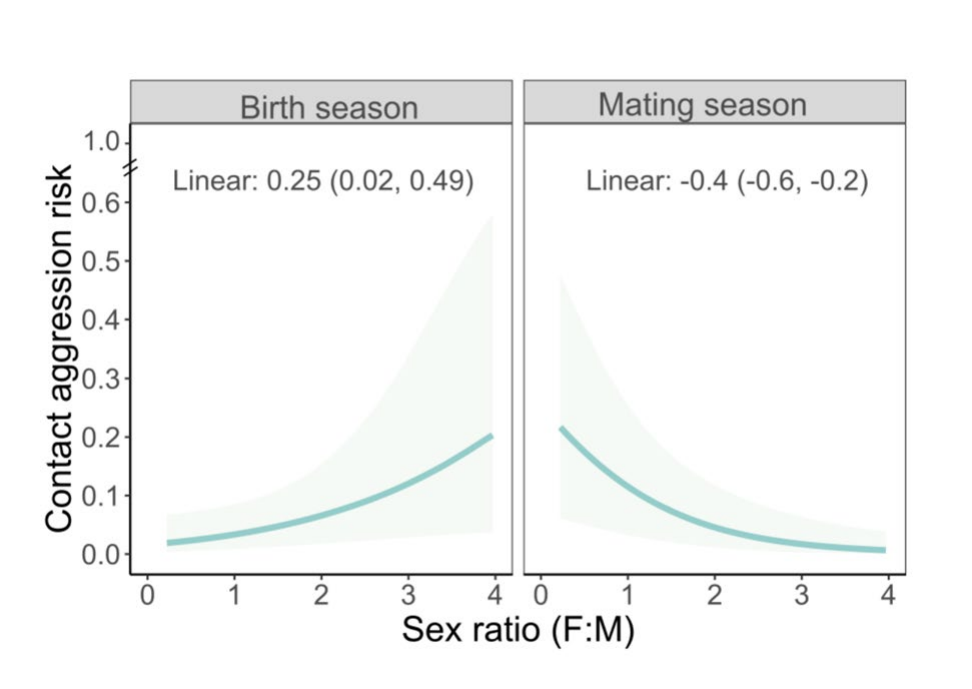
Season-dependent effect of sex ratio on the risk of male–female contact aggression. Solid lines represent the median and the shaded area the 89% CI from 100 draws of the posterior distribution. Values along the top of the plots are the median and the 89% HDPI from the posterior distribution for the variable of interest

Sex ratio predicted the risk of male–female aggression, dependent on the reproductive season (Fig. 4, Log-Odds sex ratio*season = −0.65, SE = 0.19, 89% CI = −0.96, −0.35, Table S12). Males were more likely to aggress females in male-biased groups during the mating season (marginal effect: Log-Odds mating season = −0.396, 89% HPDI = −0.61, −0.197, Fig. 1 prediction L), while during the birth season, male–female aggression risk was higher in female-biased groups (marginal effect: Log-Odds birth season = 0.251, 89% HPDI = 0.021,0.49).

## Discussion

Altogether, our results suggest that both females and males engage in physical aggression over food and mates in the Cayo Santiago population. Intrasexual aggression among females and males was higher in smaller and larger groups, consistent with the optimal group size hypothesis. Females were also more aggressive towards other females when the groups had fewer male partners available (female-biased sex ratios) but only during the birth season. Males, on the other hand, did not show changes in the rate of aggression towards other males in relation to sex ratio, as would be expected if they competed directly over females. However, males were more likely to aggress females in male-biased groups during the mating season, a pattern in line with indirect male competition over mating opportunities, where males coerce females to mate with them. Strikingly, none of the within-group aggression results aligned with the incidence of injuries, which suggests that aggression within a group may not be the main cause of injuries.

Competition for food, both within and between social groups, appears to influence aggression risk in both female and male rhesus macaques in the Cayo Santiago population. The risk of female-female aggression was higher in both smaller and larger groups, which aligns with findings in other primate species, suggesting that social groups may have an optimal size, balancing the trade-offs of competition within and between groups (Pride 2005; Markham et al. 2015; Tinsley Johnson et al. 2021). Although food provisioning is expected to reduce the incentive to use fierce aggression over food, we found higher rates of within-group aggression in smaller groups which is consistent with previous evidence in this population showing that smaller groups are more easily displaced from feeding stations due to between-group competition (Balasubramanian et al. 2014), thus leading individuals in smaller groups to compete more fiercely with one another when they do have access to food. Nevertheless, we also found higher within-group aggression in large groups, suggesting higher competition due to the reduced per capita availability of food. Despite differences in life-history strategies, male-male aggression was also higher in smaller and especially in larger groups. This suggests that male rhesus macaques also appear to compete for food, particularly in larger groups, likely in order to improve body condition for endurance during the mating season (Higham et al. 2011; Higham and Maestripieri 2014). Information on food availability and distribution is needed to clarify the drivers of intrasexual aggression in both sexes across different group sizes.

Although the risk of aggression was higher in both smaller and larger groups for both males and females, the risk of injury for females was unrelated to group size, while males in smaller groups faced the highest injury risk. This finding aligns with life-history differences between the sexes, which predict greater male involvement in between-group conflict, as males are more invested in defending territories where females are present (Trivers 1972; Smith et al. 2022). Specifically, males in smaller groups may be more frequently targeted in these between-group conflicts (Dyble et al. 2019; Smith et al. 2022). Due to the high population density on Cayo Santiago, between-group encounters are likely more common than in wild populations, potentially resulting in more frequent escalations of conflict. Moreover, male rhesus macaques are more likely to be injured when they have been for a short period in a group (Pavez-Fox et al. 2022) and also during dispersals (CMK et al., unpubl. data), suggesting that severe attacks during immigration attempts could also result in injuries. Alternatively, explanations related to our behavioral sampling regime must be considered. Rare events like aggressive interactions are often recorded using all-occurrences and ad-libitum sampling. However, we restricted our analysis to focal data to account for sampling effort, which may have led to an underestimation of the true occurrence rate. This could have caused us to miss even rarer forms of physical aggression, such as coalitionary attacks, which may be more likely to result in injuries than interactions involving a single attacker and victim. Nevertheless, underestimating aggression rates is more likely to explain a lack of a detected relationship (null effect) between aggression risk and group size, rather than the inverse or negative relationship, as observed in our study. Future research incorporating information on between-group encounters, along with more detailed information on coalitionary attacks and dispersal costs will be essential to better understand the primary cause of male injury in this population.

Regarding the relationship between sex ratio and competition, we found that when the relative availability of males in the group was lower, females had a higher risk of being aggressed by other females, but this pattern was only observed during the birth season. There are a couple potential explanations for this finding. On one hand, this might support the hypothesis that females compete for male friends. Male–female relationships during the birth season have been shown to predict the formation of temporary mating associations (i.e., consortships) in rhesus macaques (Hill 1990), suggesting that females may compete at this time in anticipation of securing future mating partners. Interestingly, previous research in this population has also found that consort pairs are more likely to tolerate each other in feeding contexts (Dubuc et al. 2012), indicating that one potential benefit for females in forming male friendships is access to food during the mating season. Male–female relationships may also confer other benefits to females, including protection against infanticide (Baniel et al. 2018a), male parental care (Baniel et al. 2018b), protection from harassment by other males (Nguyen et al. 2009), and even reduced aggression from their male friends (Haunhorst et al. 2017). Infanticide is believed to be rare in rhesus macaques (Ciani 1984), and so it is more likely that females compete for males to secure protection, reduce harassment, or prevent infant kidnapping by other females, which has been observed in this species (Maestripieri 1993). It is also plausible that females were competing for food rather than for males. The number of newborns in a group is directly related to the number of lactating females, and groups with a female-biased sex ratio may face increased energetic demands due to the cost of lactation (Dufour and Sauther 2002) heightening the incentive for both females and males to compete for food. Consistent with this idea, male–female aggression was also higher in female-biased groups during the birth season. Future studies that consider male–female affiliative interactions along with female body condition and the availability of food resources may help clarify whether competition for male friends or for food is the primary driver of contact aggression among females in this population during the birth season.

We did not find evidence that male rhesus macaques directly compete for females. Male-male aggression and male injury risk were not predicted by the sex ratio, regardless of the reproductive season, suggesting that males primarily compete indirectly. This finding aligns with the notion that sperm competition, endurance rivalry, and sneaky copulation are common mating strategies in male rhesus macaques (Higham et al. 2011; Higham and Maestripieri 2014). Furthermore, male traits typically associated with contest competition, such as body size and canine length, do not predict the winner in dyadic agonistic interactions in this species (Kimock et al. 2022). In support of alternative mating strategies, we found evidence for male–female coercion. As predicted, the risk of male–female aggression was higher in groups with a lower relative abundance of females. In mammals, males often resort to coercive tactics when there is a high degree of sexual size dimorphism and when females exert mate choice or resist mating attempts. In such contexts, males may use intimidation to force copulations or prevent females from mating with male competitors (Davidian et al. 2022). Although rhesus macaques exhibit relatively low sexual size dimorphism, both female mate choice and male coercion have been documented in this species (Manson 1992, 1994). Male–female aggression in rhesus macaques is typically initiated by high-ranking males attempting to herd females, preventing them from copulating with lower-ranking and out-group males (Berard et al. 1994). In fact, a proposed explanation for the formation of male–female affiliative relationships is to reduce the aggression of high-ranking males (Manson 1992), a hypothesis supported by evidence showing that high-status males tend to have more female affiliates (Hill 1990).

Although we found no evidence that group size or sex ratio predicted female injury risk, recent research in this population has shown that female injuries do occur, with their likelihood influenced by factors such as social status, age, and the number of female relatives in the group (Pavez-Fox et al. 2022). If it is the case that injuries primarily occur during between-group encounters, this raises important questions about how individual and group level attributes interact to shape females’ incentives for participating in between-group competition. While injuries have been linked to reduced lifespans in both males and females in this population (Pavez-Fox et al. 2022), the ad libitum nature of our injury data and the use of focal sampling to detect aggressive interactions may have led to an underestimation of both injury frequency and its connection to within-group competition. More systematic data collection on injuries and more targeted recording of aggressive events (e.g., all occurrence sampling) would provide a clearer understanding of the fitness costs associated with both within-group and between-group aggression.

Overall, this study enhances our understanding of how group size and sex ratio shape animal societies by empirically testing a range of socioecological hypotheses in a predator-free system, allowing us to directly link injuries to conspecific aggression. Despite food provisioning in this population, we found evidence supporting optimal group sizes that minimize within-group aggression for both sexes. These results align with hypotheses suggesting that both within- and between-group competition for food likely impose costs on animal societies. However, we lack data on between-group interactions or food availability, preventing us from explicitly testing the drivers of these relationships, which remains a key area for future investigation. Additionally, we linked variations in group size to male injury risk, offering a clearer understanding of fitness costs of competition. Yet, this finding did not fully align with our aggression results, raising new questions about the pathways through which group size affects injury risk. As expected for rhesus macaques, we found that sex ratio predicted the occurrence of sexual conflict (i.e., male coercion of females). Moreover, sex ratio predicted aggression not only during the mating season but also during the birth season, potentially driven by female competition for male friends or food resources. Future studies incorporating data on food availability during reproductive periods of high energetic demand, as well as information on intersexual affiliative interactions, are needed to better clarify the incentives for inter and intrasexual aggression in rhesus macaques.

## Supplementary Information

Below is the link to the electronic supplementary material.

Supplementary file1 (DOCX 760 KB)

## Data Availability

Data used in the analyses is available at Mendeley data:10.17632/nhyvdhrcw8.1

## Code availability

R code used for models and plots available at https://github.com/MPavFox/Socioecological-drivers-of-injuries.git
