# Supplementary material for: Socioecological drivers of injuries and aggression in female and male rhesus macaques (Macaca mulatta)
Source: Behav Ecol Sociobiol. 2025 Mar 28;79(3):47. doi: 10.1007/s00265-025-03587-3 (PMC11953099; doi:10.1007/s00265-025-03587-3)
Supplement: Supplementary file 1 — Supplementary file1 (DOCX 760 KB) [file 265_2025_3587_MOESM1_ESM.docx]

**Electronic Supplementary Material**

**Supplement to:** Socioecological drivers of injuries and aggression in female and male rhesus macaques (*Macaca mulatta*)

**Journal:** Behavioral Ecology and Sociobiology

**Authors:** Melissa A. Pavez-Fox^1,2,*^, Erin R. Siracusa^1^, Samuel Ellis^1^, Clare M. Kimock^3,4^, Nahiri Rivera-Barreto^5^, Josue E. Negron-Del Valle^6^, Daniel Phillips^6^, Angelina Ruiz-Lambides^5^, Noah Snyder-Mackler^6,7,8^, James P. Higham^3^, Delphine De Moor^1,†^, and Lauren J.N. Brent^1,†^

.

**Affiliations:**

^1^Centre for Research in Animal Behaviour, University of Exeter, Exeter, United Kingdom EX4 4QG

^2^Department of Psychology and Neuroscience, University of St Andrews, United

Kingdom KY16 9JP

^3^Department of Anthropology, New York University, New York, NY 10003

^4^Department of Psychology, Nottingham Trent University, Nottingham, United Kingdom NG1 4FQ

^5^Caribbean Primate Research Center, University of Puerto Rico, San Juan, Puerto Rico 00936-5067

^6^Center for Evolution and Medicine, Arizona State University, Temple, AZ 85281, United States

^7^School of Life Sciences, Arizona State University, Temple, AZ 85281

^8^School for Human Evolution and Social Change, Arizona State University, Temple, AZ 85281

^†^Equal contribution

^*^Corresponding author: Melissa Andrea Pavez Fox

Email: melissa.pavez.fox@gmail.com

**Table S1.** Output from logistic model predicting female injury risk as a quadratic function of the number of females in the group and the interaction between sex ratio (females-to-males) and reproductive season.

|  | **Injury risk** | | |
| --- | --- | --- | --- |
| *Predictors* | *Log-Odds* | *std. Error* | *CI (89%)* |
| Intercept | -4.40 | 0.17 | -4.70 – -4.13 |
| scaled_fem | 0.03 | 0.07 | -0.09 – 0.14 |
| Iscaled_femE2 | -0.07 | 0.06 | -0.18 – 0.03 |
| scaled_SR | -0.04 | 0.09 | -0.19 – 0.10 |
| is_mating: is_mating1 | 0.97 | 0.23 | 0.60 – 1.36 |
| scaled_SR:is_mating1 | 0.08 | 0.11 | -0.10 – 0.26 |
| N _id_ | 792 | | |
| N _year_bim_ | 46 | | |
| Observations | 15952 | | |

**Iscaled_femE2** = quadratic term for number of females, **scaled_fem** = z-standardised number of females, **scaled_SR =** z-standardised sex ratio, **is_mating: is_mating1 =** mating season. Individual ID (**id**) and bimonthly interval (**year_bim**) were included as random effects.

**Table S2.** Output from logistic model predicting female injury risk as a quadratic function of the number of females in the group and the sex ratio (females-to-males).

|  | **Injury risk** | | |
| --- | --- | --- | --- |
| *Predictors* | *Log-Odds* | *std. Error* | *CI (89%)* |
| Intercept | -4.41 | 0.18 | -4.70 – -4.13 |
| scaled_fem | 0.03 | 0.07 | -0.09 – 0.15 |
| Iscaled_femE2 | -0.07 | 0.06 | -0.17 – 0.03 |
| scaled_SR | 0.01 | 0.06 | -0.09 – 0.10 |
| is_mating: is_mating1 | 0.99 | 0.23 | 0.60 – 1.37 |
| N _id_ | 792 | | |
| N _year_bim_ | 46 | | |
| Observations | 15952 | | |

**Iscaled_femE2** = quadratic term for number of females, **scaled_fem** = z-standardised number of females, **scaled_SR =** z-standardised sex ratio, **is_mating: is_mating1 =** mating season. Individual ID (**id**) and bimonthly interval (**year_bim**) were included as random effects.

**Table S3.** Output from logistic model predicting female injury risk as a linear function of the number of females in the group and sex ratio (females-to-males).

|  | **Injury risk** | | |
| --- | --- | --- | --- |
| *Predictors* | *Log-Odds* | *std. Error* | *CI (89%)* |
| Intercept | -4.48 | 0.17 | -4.76 – -4.22 |
| scaled_fem | 0.04 | 0.07 | -0.07 – 0.15 |
| scaled_SR | 0.01 | 0.06 | -0.08 – 0.10 |
| is_mating: is_mating1 | 1.00 | 0.24 | 0.61 – 1.39 |
| N _id_ | 792 | | |
| N _year_bim_ | 46 | | |
| Observations | 15952 | | |

**scaled_fem** = z-standardised group size, **scaled_SR =** z-standardised sex ratio, **is_mating: is_mating1 =** mating season. Individual ID (**id**) and bimonthly interval (**year_bim**) were included as random effects.

**Table S4.** Output from logistic model predicting female-female contact aggression rates as a quadratic function of the number of females in the group and the interaction between sex ratio (females-to-males) and the reproductive season.

|  | **Risk of contact aggression** | | |
| --- | --- | --- | --- |
| *Predictors* | *Log-Odds* | *std. Error* | *CI (89%)* |
| Intercept | -4.36 | 0.21 | -4.72 – -4.04 |
| scaled_fem | 0.05 | 0.12 | -0.15 – 0.25 |
| Iscaled_femE2 | 0.20 | 0.11 | 0.01 – 0.38 |
| scaled_SR | 0.22 | 0.13 | 0.02 – 0.42 |
| is_mating: is_mating1 | 0.15 | 0.25 | -0.25 – 0.56 |
| scaled_SR:is_mating1 | -0.30 | 0.18 | -0.59 – -0.01 |
| N _id_ | 422 | | |
| N _year_bim_ | 39 | | |
| Observations | 4390 | | |

**Iscaled_femE2** = quadratic term for number of females, **scaled_fem** = z-standardised number of females, **scaled_SR =** z-standardised sex ratio, **is_mating: is_mating1 =** mating season. Individual ID (**id**) and bimonthly interval (**year_bim**) were included as random effects.

**Table S5.** Output from logistic model predicting male injury risk as a quadratic function of the number of males in the group and the interaction between sex ratio (females-to-males) and reproductive season.

|  | **Injury risk** | | |
| --- | --- | --- | --- |
| *Predictors* | *Log-Odds* | *std. Error* | *CI (89%)* |
| Intercept | -4.07 | 0.16 | -4.34 – -3.83 |
| scaled_male | -0.14 | 0.10 | -0.30 – 0.02 |
| Iscaled_maleE2 | -0.09 | 0.05 | -0.18 – -0.00 |
| scaled_SR | 0.07 | 0.10 | -0.09 – 0.22 |
| is_mating: is_mating1 | 1.36 | 0.21 | 1.02 – 1.70 |
| scaled_SR:is_mating1 | -0.06 | 0.10 | -0.23 – 0.11 |
| N _id_ | 730 | | |
| N _year_bim_ | 46 | | |
| Observations | 12984 | | |

**Iscaled_maleE2** = quadratic term for number of males, **scaled_male** = z-standardised number of males, **scaled_SR =** z-standardised sex ratio, **is_mating: is_mating1 =** mating season. Individual ID (**id**) and bimonthly interval (**year_bim**) were included as random effects.

**Table S6.** Output from logistic model predicting male injury risk as a quadratic function of the number of males in the group and a linear function of sex ratio (females-to-males).

|  | **Injury risk** | | |
| --- | --- | --- | --- |
| *Predictors* | *Log-Odds* | *std. Error* | *CI (89%)* |
| Intercept | -4.07 | 0.16 | -4.34 – -3.82 |
| scaled_male | -0.15 | 0.10 | -0.31 – 0.01 |
| Iscaled_maleE2 | -0.09 | 0.06 | -0.18 – -0.00 |
| scaled_SR | 0.03 | 0.07 | -0.08 – 0.14 |
| is_mating: is_mating1 | 1.35 | 0.21 | 1.01 – 1.70 |
| N _id_ | 730 | | |
| N _year_bim_ | 46 | | |
| Observations | 12984 | | |

**Iscaled_maleE2** = quadratic term for number of males, **scaled_male** = z-standardised number of males, **scaled_SR =** z-standardised sex ratio, **is_mating: is_mating1 =** mating season. Individual ID (**id**) and bimonthly interval (**year_bim**) were included as random effects.

**Table S7.** Output from logistic model predicting male injury risk as a linear function of the number of males in the group and sex ratio (females-to-males).

|  | **Injury risk** | | |
| --- | --- | --- | --- |
| *Predictors* | *Log-Odds* | *std. Error* | *CI (89%)* |
| Intercept | -4.16 | 0.15 | -4.41 – -3.92 |
| scaled_male | -0.22 | 0.08 | -0.36 – -0.09 |
| scaled_SR | 0.00 | 0.06 | -0.10 – 0.10 |
| is_mating: is_mating1 | 1.35 | 0.21 | 1.01 – 1.69 |
| N _id_ | 730 | | |
| N _year_bim_ | 46 | | |
| Observations | 12984 | | |

**Scaled_male** = z-standardised number of males, **scaled_SR =** z-standardised sex ratio, **is_mating: is_mating1 =** mating season. Individual ID (**id**) and bimonthly interval (**year_bim**) were included as random effects.

**Table S8.** Output from logistic model predicting male-male contact aggression rates as a quadratic function of the number of males in the group and the interaction between sex ratio (females-to-males) and the reproductive season.

|  | **Risk of contact aggression** | | |
| --- | --- | --- | --- |
| *Predictors* | *Log-Odds* | *std. Error* | *CI (89%)* |
| Intercept | -4.99 | 0.24 | -5.42 – -4.63 |
| scaled_male | 0.10 | 0.20 | -0.22 – 0.43 |
| Iscaled_maleE2 | 0.12 | 0.08 | -0.01 – 0.24 |
| scaled_SR | -0.01 | 0.24 | -0.39 – 0.37 |
| is_mating: is_mating1 | 0.56 | 0.28 | 0.10 – 1.00 |
| scaled_SR:is_mating1 | 0.15 | 0.23 | -0.22 – 0.52 |
| N _id_ | 326 | | |
| N _year_bim_ | 39 | | |
| Observations | 3154 | | |

**Iscaled_maleE2** = quadratic term for number of males, **scaled_male** = z-standardised number of males, **scaled_SR =** z-standardised sex ratio, **is_mating: is_mating1 =** mating season. Individual ID (**id**) and bimonthly interval (**year_bim**) were included as random effects.

**Table S9.** Output from logistic model predicting male-male contact aggression rates as a quadratic function of the number of males in the group and a linear function of the sex ratio (females-to-males).

|  | **Risk of contact aggression** | | |
| --- | --- | --- | --- |
| *Predictors* | *Log-Odds* | *std. Error* | *CI (89%)* |
| Intercept | -5.00 | 0.24 | -5.42 – -4.64 |
| scaled_male | 0.11 | 0.20 | -0.21 – 0.44 |
| Iscaled_maleE2 | 0.13 | 0.08 | 0.00 – 0.25 |
| scaled_SR | 0.08 | 0.20 | -0.24 – 0.40 |
| is_mating: is_mating1 | 0.54 | 0.27 | 0.09 – 0.98 |
| N _id_ | 326 | | |
| N _year_bim_ | 39 | | |
| Observations | 3154 | | |

**Iscaled_maleE2** = quadratic term for number of males, **scaled_male** = z-standardised number of males, **scaled_SR =** z-standardised sex ratio, **is_mating: is_mating1 =** mating season. Individual ID (**id**) and bimonthly interval (**year_bim**) were included as random effects.

**Table S10.** Output from logistic model predicting male-male contact aggression rates as a linear function of the number of males in the group and the sex ratio (females-to-males).

|  | **Risk of contact aggression** | | |
| --- | --- | --- | --- |
| *Predictors* | *Log-Odds* | *std. Error* | *CI (89%)* |
| Intercept | -4.86 | 0.23 | -5.28 – -4.53 |
| scaled_male | 0.11 | 0.21 | -0.22 – 0.44 |
| scaled_SR | 0.09 | 0.20 | -0.22 – 0.42 |
| is_mating: is_mating1 | 0.54 | 0.29 | 0.07 – 1.02 |
| N _id_ | 326 | | |
| N _year_bim_ | 39 | | |
| Observations | 3154 | | |

**Scaled_male** = z-standardised number of males, **scaled_SR =** z-standardised sex ratio, **is_mating: is_mating1 =** mating season. Individual ID (**id**) and bimonthly interval (**year_bim**) were included as random effects.

**Table S11.** Output from logistic model predicting risk of male-to-female contact aggression as a quadratic function of group size and the interaction between sex ratio (females-to-males) and the reproductive season.

|  | **Risk of contact aggression** | | |
| --- | --- | --- | --- |
| *Predictors* | *Log-Odds* | *std. Error* | *CI (89%)* |
| Intercept | -4.49 | 0.19 | -4.81 – -4.19 |
| Iscaled_groupE2 | -0.01 | 0.06 | -0.10 – 0.08 |
| scaled_group | -0.12 | 0.11 | -0.29 – 0.05 |
| scaled_SR | 0.26 | 0.15 | 0.02 – 0.50 |
| is_mating: is_mating1 | 0.67 | 0.25 | 0.27 – 1.08 |
| scaled_SR:is_mating1 | -0.65 | 0.20 | -0.96 – -0.32 |
| N _id_ | 422 | | |
| N _year_bim_ | 39 | | |
| Observations | 4390 | | |

**Iscaled_groupE2** = quadratic term for group size, **scaled_group** = z-standardised group size, **scaled_SR =** z-standardised sex ratio, **is_mating: is_mating1 =** mating season. Individual ID (**id**) and bimonthly interval (**year_bim**) were included as random effects.

**Table S12.** Output from logistic model predicting risk of male-to-female contact aggression as a linear function of group size and the interaction between sex ratio (females-to-males) and the reproductive season.

|  | **Risk of contact aggression** | | |
| --- | --- | --- | --- |
| *Predictors* | *Log-Odds* | *std. Error* | *CI (89%)* |
| Intercept | -4.48 | 0.19 | -4.81 – -4.20 |
| scaled_group | -0.12 | 0.08 | -0.25 – 0.01 |
| scaled_SR | 0.25 | 0.15 | 0.02 – 0.49 |
| is_mating: is_mating1 | 0.67 | 0.24 | 0.27 – 1.06 |
| scaled_SR:is_mating1 | -0.65 | 0.19 | -0.96 – -0.35 |
| N _id_ | 422 | | |
| N _year_bim_ | 39 | | |
| Observations | 4390 | | |

**scaled_group** = z-standardised group size, **scaled_SR =** z-standardised sex ratio, **is_mating: is_mating1 =** mating season. Individual ID (**id**) and bimonthly interval (**year_bim**) were included as random effects.

# Supplementary Figures


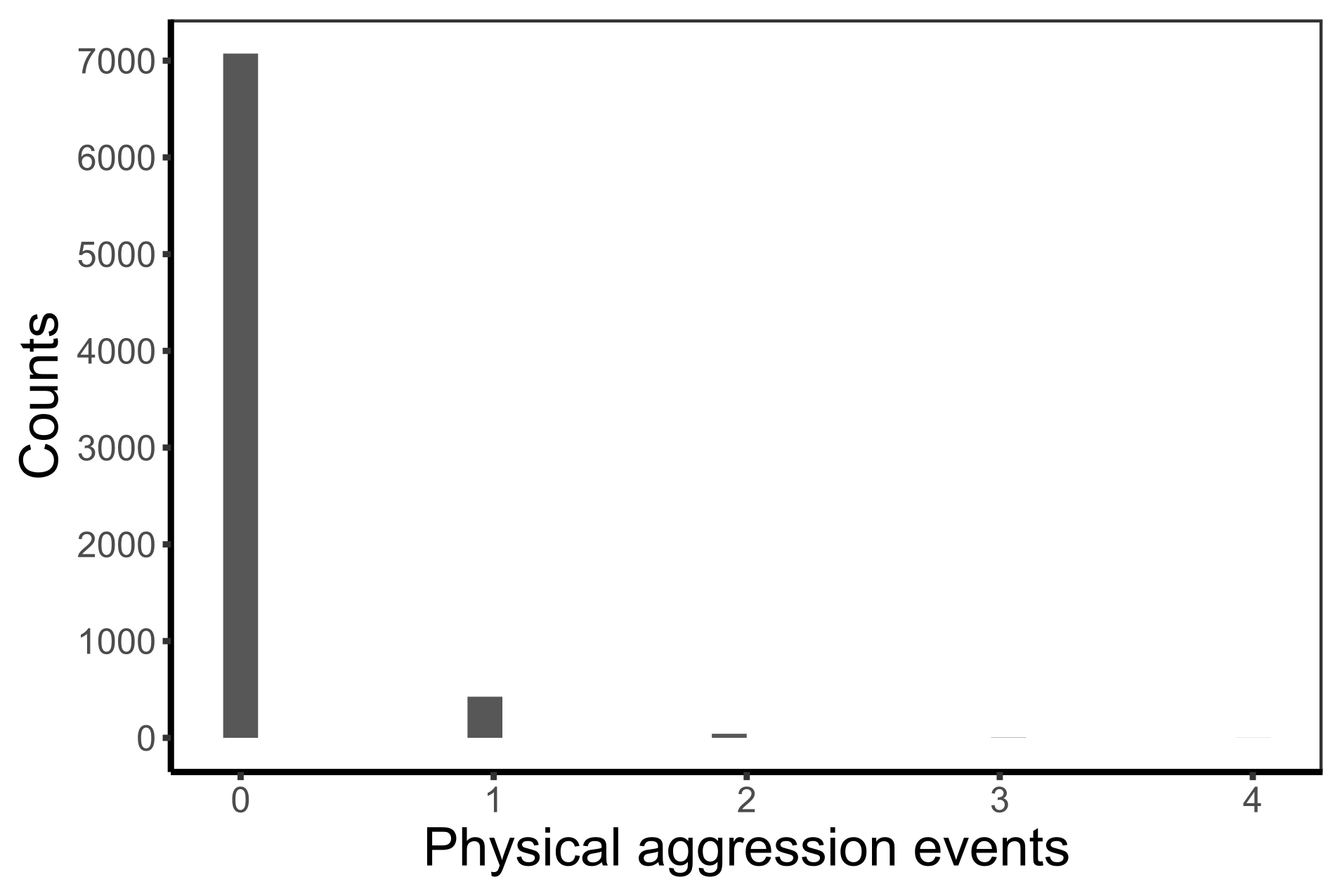


Figure S1: **Histogram of physical aggression received by a focal animal in a given bimonthly interval across the study period.**


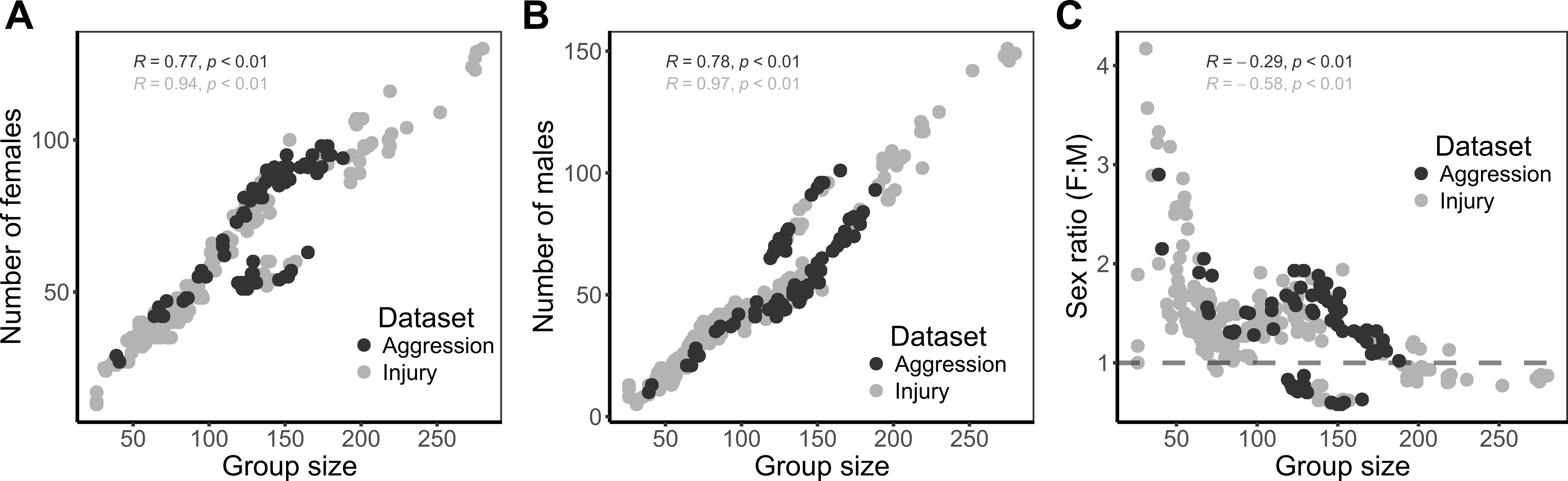


Figure S2: **Correlations of A) number of females, B) number of males and C) adult sex ratio with group size.**


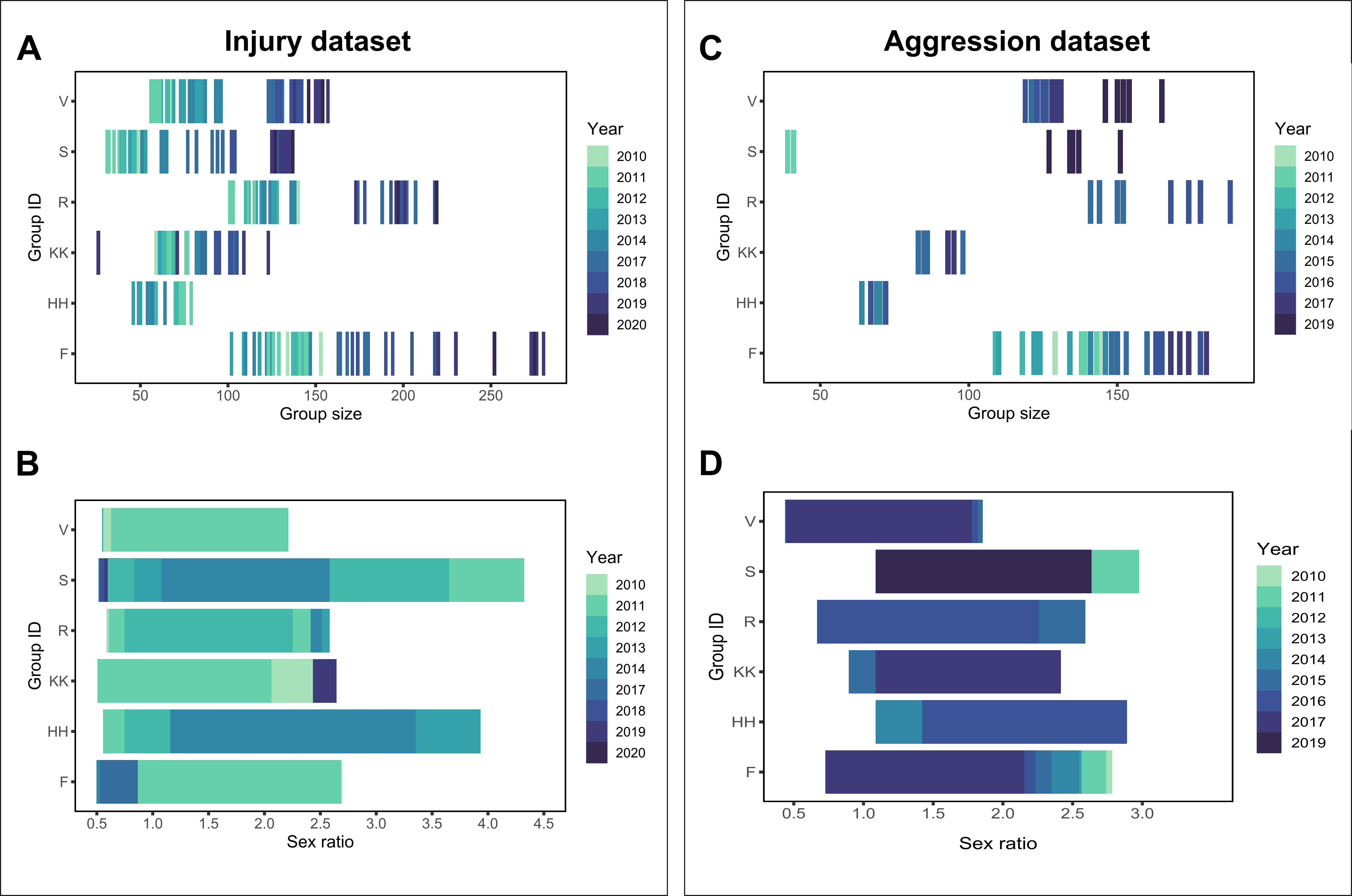


Figure S3: **Variation of group size and operational sex ratio per behavioural group across the 10 years of study.**
